# Insights into Structural and Mechanistic Features of Viral IRES Elements

**DOI:** 10.3389/fmicb.2017.02629

**Published:** 2018-01-04

**Authors:** Encarnacion Martinez-Salas, Rosario Francisco-Velilla, Javier Fernandez-Chamorro, Azman M. Embarek

**Affiliations:** Centro de Biología Molecular Severo Ochoa, Consejo Superior de Investigaciones Científicas – Universidad Autónoma de Madrid, Madrid, Spain

**Keywords:** RNA viruses, IRES elements, conserved RNA motifs, RNA structure, RNA-binding proteins

## Abstract

Internal ribosome entry site (IRES) elements are *cis*-acting RNA regions that promote internal initiation of protein synthesis using cap-independent mechanisms. However, distinct types of IRES elements present in the genome of various RNA viruses perform the same function despite lacking conservation of sequence and secondary RNA structure. Likewise, IRES elements differ in host factor requirement to recruit the ribosomal subunits. In spite of this diversity, evolutionarily conserved motifs in each family of RNA viruses preserve sequences impacting on RNA structure and RNA–protein interactions important for IRES activity. Indeed, IRES elements adopting remarkable different structural organizations contain RNA structural motifs that play an essential role in recruiting ribosomes, initiation factors and/or RNA-binding proteins using different mechanisms. Therefore, given that a universal IRES motif remains elusive, it is critical to understand how diverse structural motifs deliver functions relevant for IRES activity. This will be useful for understanding the molecular mechanisms beyond cap-independent translation, as well as the evolutionary history of these regulatory elements. Moreover, it could improve the accuracy to predict IRES-like motifs hidden in genome sequences. This review summarizes recent advances on the diversity and biological relevance of RNA structural motifs for viral IRES elements.

## Introduction

**FIGURE 1:**
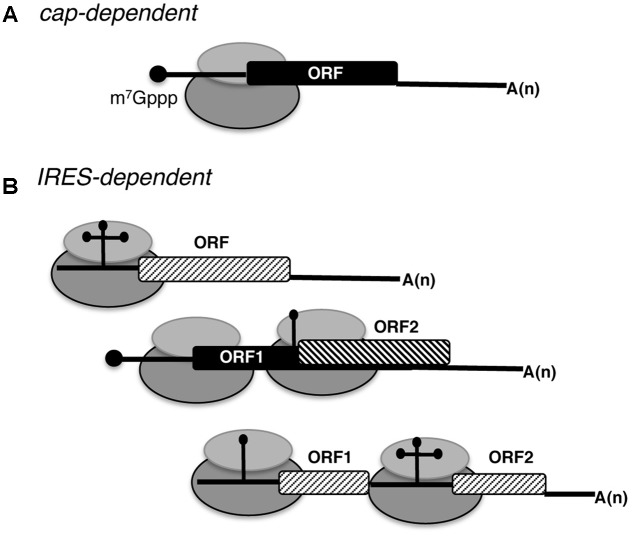
Schematic representation of eukaryotic mRNAs. **(A)** Basic features of a conventional monocistronic mRNA translated via a cap-dependent mechanism. The black circle at the 5′ end depicts the cap (m^7^Gppp). The ribosome is represented by gray filled ovals; the black filled rectangle depicts the open reading frame (ORF), and A(n) the poly(A) tail. **(B)** Distinct types of mRNAs translated using IRES-dependent mechanisms. RNA regions adopting different stem-loop structures promote internal initiation of translation; the cap at the 5′ end and the poly(A) tail at the 3′ end can be present or absent. Monocistronic and dicistronic mRNAs (containing overlapping ORFs or independent ORFs) are depicted.

Regulation of protein synthesis is a key step of gene expression in all organisms. The process of RNA translation occurs in four basic steps, initiation, elongation, termination, and ribosome recycling. In eukaryotes, the vast majority of mRNAs initiate translation by a cap-dependent mechanism (Hinnebusch, 2014). This general mechanism depends on the recognition of the m^7^G(5′)ppp(5′)N (designated cap) structure placed at the 5′ end of most mRNAs (**Figure 1A**) by the translation eukaryotic initiation factor (eIF)-4F, which is composed of three polypeptides (eIF4A, eIF4E, and eIF4G). In addition, eIF4F mediates the assembly of the 43S preinitiation complex, which consists of the 40S ribosomal subunit bound to the multi-subunit factor eIF3, and the ternary complex (composed of eIF2-GTP and the initiator Met-tRNAi). The 43S complex scans the 5′ untranslated region (UTR) of the mRNA in 5′–3′ direction until an AUG triplet is found in the appropriate context to start protein synthesis. Upon start codon recognition via base pairing of Met-tRNAi with mRNA in the P site of the ribosome, conformational rearrangements trigger the formation of the 48S preinitiation complex. In this step, eIF5B displaces eIF2-GDP from Met-tRNAi promoting 60S ribosomal subunit joining together with eIF1A, leading to the formation of the elongation competent 80S ribosome.

Beyond this general manner to initiate translation, under certain physiological conditions specific mRNAs initiate protein synthesis using a cap-independent mechanism, which eventually could lead to the synthesis of a different polypeptide. Research conducted to understand translation control operating in specific mRNAs provided evidence for different cap-independent mechanisms (Du et al., 2013; Coots et al., 2017). One of the cap-independent mechanisms is exemplified by internal ribosome entry site (IRES) elements (**Figure 1B**).

It is widely established that strong cellular stresses, such as viral infection, compromise cap-dependent translation initiation. Yet, viral mRNAs overcome the inhibitory conditions using various strategies (Walsh and Mohr, 2011; Simon and Miller, 2013). One of these strategies is exemplified by the initiation mechanism driven by mRNA regions referred to as IRES elements (**Figure 1B**), first reported in the genomic RNA of picornaviruses (Jang et al., 1988; Pelletier and Sonenberg, 1988).

IRES elements are RNA regions that recruit the 40S ribosomal subunit through cap-independent mechanisms. These elements often adopt complex RNA structures, which serve as the anchoring site for the ribosome guided by RNA–RNA and/or RNA–protein interactions. Various RNA viruses that have a positive-strand uncapped genome depend on IRES elements to govern viral protein synthesis. Accordingly, IRES elements promote translation initiation of hepacivirus, pestivirus, and dicistrovirus genomic RNAs, among others (Lee et al., 2017). Viral IRES elements hijack the translational machinery of the host cell; these RNA regulatory elements promote translation initiation internally by recruiting and actively manipulating the ribosome, in most cases using a subset of canonical eIFs and cellular RNA-binding proteins (RBPs).

Regarding their position on the mRNA, IRES elements are generally located within the 5′ UTR, although a few examples of viral and cellular IRES elements placed within the coding sequence (**Figure 1B**) have been described (Locker et al., 2011; Karginov et al., 2016). This is currently an unpredictable feature, which, however, greatly increases the genome coding potential. Hence, it needs to be taken into consideration in order to fix the annotation of genomes.

A key feature of viral IRES elements is that they are autonomous elements. This means that they are active outside of its natural RNA context. However, a prominent characteristic of viral IRES elements is that they function as a single entity, e.g., short regions of the element do not exhibit the activity produced by the entire element. This property occurs in spite of their modular RNA structure organization, which in fact allows a distribution of functions among their modular domains (Lozano and Martinez-Salas, 2015). Nevertheless, the multidomain organization of IRES elements could be relevant to understand the function, and also the evolutionary history, of their conserved RNA structural motifs.

This review is focused on the structural features of viral IRES that have an impact on the mechanism of internal translation initiation.

## Internal Initiation Mechanisms: Rna Structural Motifs Involved in Ires Function

RNA structure determines the function of the vast majority of viral IRES elements. However, although the IRES elements present in the genome of RNA viruses perform a similar function, a universal IRES structural motif remains elusive. In fact, IRES elements present in the genome of different families of RNA viruses lack overall conserved features (Martinez-Salas et al., 2015). For instance, well-established IRES elements, such as the intergenic region (IGR) of dicistroviruses, the IRES of hepatitis C virus (HCV) and those of picornaviruses, which belong to different families of RNA viruses, lack sequence homology and exhibit different structural organization. These IRES elements also differ in the requirement of factors to assemble 48S initiation complexes. Nonetheless, the natural selection pressure has evolved specialized three-dimensional structures in each family of RNA virus rendering diverse functional elements able to govern initiation of protein synthesis. Indeed, the RNA architecture of the IGR is strongly conserved across different species of dicistroviruses (Nakashima and Uchiumi, 2009). In contrast, the 5′ end IRES and the IGR are different in dicistroviruses themselves. On the other hand, the encephalomyocarditis virus (EMCV) and foot-and-mouth disease virus (FMDV) picornavirus IRES elements display similar secondary structures although they differ in 50% of their sequence, and to a large extent use the same mechanism to recruit ribosomal subunits (Lozano et al., 2016a). Further supporting the role of RNA structure for IRES function, the secondary structure of highly variable RNA viruses, such as HCV and FMDV, is preserved by compensatory mutations (Honda et al., 1999; Lozano and Martinez-Salas, 2015). In accordance with this observation, engineered disruption of stems and conserved motifs in loops and internal bulges drastically reduce IRES activity, whereas compensatory mutations restoring the secondary structure recover translation efficiency (Honda et al., 1996; Fernandez et al., 2011a).

Viral IRES elements have been classified into different types, which are related to their structural organization and, in turn, to their mechanism of initiation. Basically, there are two different manners to promote internal initiation of translation. The simplest one occurs by direct interaction of the IRES with the 40S ribosomal subunit. A more complex and frequent manner occurs by binding of the IRES element to eIFs and RBPs, which contribute to recruit the 40S ribosomal subunit. For instance, the dicistrovirus IGR or the HCV IRES physically associate the 40S subunit (Yamamoto et al., 2017). Nevertheless, there are notable differences among these viral IRES elements. Whereas the IGR assembles 48S initiation complexes *in vitro* in the absence of eIFs (Wilson et al., 2000), the IRES element of HCV require eIFs to assemble 48S complexes in reconstitution assays (Pestova et al., 1998).

### Dicistrovirus IRES Elements

**FIGURE 2:**
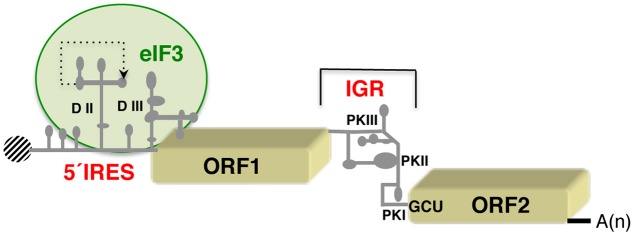
Schematic representation of the dicistrovirus RNA genome. The IRES on the 5′ UTR of the cricket paralysis virus (CrPV) RNA and the intergenic region (IGR) promoting translation of the open reading frames (ORF1 and ORF2, respectively) are indicated. Gray lines depict domains (DII, DIII) and pseudoknots (PKI, II, III) referred to as in the text. A dashed-line indicates the tertiary interaction predicted in domain II of the 5′ end IRES. A green circle denotes the region involved in interaction with the multi-subunit initiation factor eIF3. A black shaded circle depicts the viral protein (Vpg) covalently linked to the 5′ end of the genome; A(n) denotes the poly(A) tract at the 3′ end.

The genome of dicistroviruses is an example of natural dicistronic mRNAs in which translation of each open reading frame (ORF) is governed by distinct IRES elements (**Figure 2**), probably because two ORFs must be separately regulated in dicistrovirus infection. The structural features of the 5′ end IRES governing translation of ORF1 differ between members of the dicistrovirus genus (Groppelli et al., 2007; Gross et al., 2017). In contrast, the overall structural organization of the IRES element located in the IGR, which drives translation of ORF2, is conserved. Regarding factors required for activity, the IGR of dicistroviruses represents the simplest category of viral IRES elements. Because of their simple mechanism for recruiting the ribosome, the IGR elements have provided not only a useful tool to understand IRES–ribosome interaction, but also to study the conformational changes occurring on the ribosome during translocation and elongation events.

The dicistrovirus IGR (about 190 nt) adopts a compact three-dimensional structure, which is essential for IRES activity (Kanamori and Nakashima, 2001). It consists of a triple-pseudoknot (PKI, II, and III; Costantino and Kieft, 2005) that functionally substitutes for the initiator met-tRNA_i_ during internal initiation, directing translation initiation at a non-AUG triplet (**Figure 2**). High-resolution studies of the three-dimensional structure of IGR–ribosome complexes showed that the PKI of the cricket paralysis virus (CrPV) IGR resembles a tRNA/mRNA interaction in the decoding center of the A site, blocking tRNA binding, and mimicking a pre-translocation rather than initiation state of the ribosome (Fernandez et al., 2014). PKII is involved in 60S association, while PKIII appears to be important for both 40S and 60S recruitment. Pseudo-translocation of the IGR by eukaryotic elongation factor 2 (eEF2) in the absence of peptide bond formation brings the first codon of the mRNA into the aminoacyl (A) site to start translation; during this translocation event, the IGR undergoes a structural change to a stretched conformation (Muhs et al., 2015). Yet, subtle differences exist on the IGR among dicistrovirus genus. For instance initiation of protein synthesis promoted by the honey bee Israeli acute paralysis virus (IAPHV) IGR can occur at two alternative frames, 0 and +1, depending on the RNA structure of the PKI domain (Butcher and Jan, 2016). These data suggest that the reading frame is established downstream of the PKI binding in the A site.

Near atomic-resolution studies of the structural conformation of the dicistrovirus IGR IRES assembled on ribosome complexes illustrate the active role of the RNA structure in manipulating the ribosome. Structural analyses of the ribosome-bound Taura syndrome virus (TSV) IRES revealed that PKI occupies the ribosomal decoding center at the A site resembling the tRNA anticodon–mRNA codon interaction. However, in contrast to conventional mRNA, the ORF of the IGR-driven mRNA is placed in the A site, whereas the 40S peptidyl (P) site remains unoccupied (Koh et al., 2014). More recently, studies on this IRES provided new insights into the internal initiation mechanism: the inchworm-like movement of the TSV IGR assembled with the ribosome and eEF2⋅GTP bound with sordarin suggests that this mRNA suffers cyclic conformational changes from extended to bent conformations coupled with ribosomal inter-subunit rotation and 40S head swivel. In addition, eEF2 attached to the 60S subunit slides along the rotating 40S subunit to enter the A site. Moreover, the eEF2 diphthamide tip at domain IV (a post-translational modification of a eEF2 histidine residue involved in translocation) separates the tRNA–mRNA-like PKI of the IGR from the decoding center, stabilizing it in a conformation reminiscent of a hybrid tRNA state (Abeyrathne et al., 2016).

Recent data concerning the dicistrovirus 5′ IRES structural organization has shown the presence of a pseudoknot relevant for translation initiation (Gross et al., 2017). Moreover, in contrast to the IGR, the 5′ IRES interacts with several eIF3 subunits (**Figure 2**). Why the 5′ UTR IRES elements of dicistroviruses are more variable than the IGRs remains elusive. Yet, the strong conservation of the IGR structure shows that its function intimately relies on a defined three-dimensional structure. Instead, the function of the 5′ UTR IRES is determined by the concerted action of the RNA conformation and the interaction with eIF3 (Gross et al., 2017). The combination of these features may allow higher RNA structure flexibility.

### Hepacivirus IRES

**FIGURE 3:**
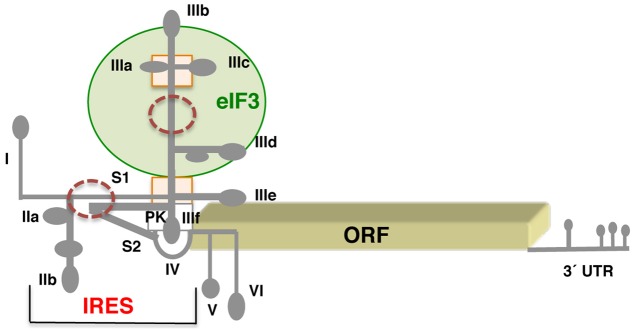
Schematic representation of the hepatitis C genome. Secondary structure of the HCV virus IRES flanked by stem loop I at the 5′ end, and hairpins V and VI at the 3′ end. Subdomains IIa,b, IIIa,b,c,d,e, and domain IV of the IRES are indicated. S1 and S2 denote short stems surrounding the pseudoknot (PK). A green circle denotes the domain III region involved in eIF3 interaction; a rectangle depicts a four-way junction in domain III; brown circles denote regions of local flexibility. The 3′ UTR contains several stem-loops, but is not polyadenylated.

The hepacivirus IRES requires eIF3 to promote translation (Pestova et al., 1998). Research conducted on the mechanism of action of the HCV IRES has shown how RNA structure may at least in part substitute for protein-based factors, as it occurs in the dicistrovirus IGR. The HCV IRES is located within the 5′ UTR of the viral genome, downstream of domain I. The spacer separating domain I from the IRES element harbors binding-sites for miR122, which are mainly involved in mRNA stability (Sarnow and Sagan, 2016). The IRES region encompasses 340 nt organized into three domains, designated as II, III, and IV (**Figure 3**). Each of these domains performs a distinct function during internal initiation. Domain II, which adopts an L-shape structure consisting of subdomains IIa and IIb, is involved in eIF5-induced GTP hydrolysis of eIF2. In addition, the apical loop of subdomain IIb contacts the ribosomal proteins RPS5, RPS7, and RPS11 (Fuchs et al., 2015; Quade et al., 2015). Domains II and III are connected by a short stem (S1). Domain III consists of a multi-bulged structure organized in subdomains IIIa, b, c, d, e, and f. A four-way junction holds the apical stem-loops IIIa, b, c. Subdomain IIIb binds eIF3, whereas IIIe binds the 40S ribosomal subunit (Filbin et al., 2013; Matsuda and Mauro, 2014). The GGG motif at the loop of subdomain IIId contacts the backbone and bases of the CCC triplet in the 18S rRNA, inducing a rearrangement of the 18S rRNA structure near the conserved rRNA nucleotide G1639 (Malygin et al., 2013). Consistent with this, independent studies of HCV–ribosome binary complexes noticed conformational changes suggesting the formation of a kissing complex between the loop IIId of the HCV IRES and the 18S rRNA (Angulo et al., 2015). At the basal region of domain III, a four-way junction connects IIIe and IIIf stem-loops; the later forms a PK structure with the short stem S2 (Berry et al., 2010). The functional start codon of the HCV RNA is placed at the loop of domain IV (Honda et al., 1996).

In solution, the HCV IRES adopts a flexible RNA structure composed of an ensemble of conformers made of rigid parts that can move relative to each other (Perard et al., 2013). The flexibility of the HCV free RNA in solution was also observed on longer transcripts harboring the IRES element flanked by domain I at the 5′ end, and domains V and VI at the 3′ end (Garcia-Sacristan et al., 2015). Further revealing the dynamic conformational features of the IRES–ribosome complex, the RNA flexibility of the HCV IRES was supported by the presence of four subpopulations for the 80S HCV IRES complex (Yamamoto et al., 2015).

Assembly of 48S initiation complexes *in vitro* with the HCV IRES, and the so-called HCV-like present in the genomic RNA of pestiviruses and some picornaviruses (Asnani et al., 2015), requires eIF3 and the ternary complex eIF2–GTP–tRNA_i_. During initiation, the IRES–40S complex places the start codon into the P site, base-pairing with eIF2-bound initiator met-tRNAi to form a 48S complex. Reconstitution of a 40S ribosomal complex containing eIF3 and a pestivirus HCV-like IRES shows that eIF3 is displaced from its ribosomal position in the 43S complex since it shares the same ribosomal binding site. Instead, it interacts through its ribosome-binding surface with the apical region of domain III of the IRES (Hashem et al., 2013). Hence, the HCV-like IRES capacity to prevent ribosomal association of eIF3 could favor translation of viral mRNAs.

It is interesting to note that the multi-subunit factor eIF3 can perform different roles during internal initiation, acting as a bridge for other eIFs and ribosomal proteins. Indeed, several eIF3 subunits could provide a functional bridge for HCV-like IRES elements with the ribosomal subunit (Boehringer et al., 2005; Gross et al., 2017). Along this idea, a recent study reported the requirement of the 40S ribosomal protein receptor of activated protein kinase C 1 (RACK1) for both, the IGR of CrPV and HCV IRES-dependent translation (Majzoub et al., 2014), although this protein maps to different sites on 80S ribosome complexes assembled with CrPV or HCV IRES elements. The interaction of RACK1 with the HCV IRES seems to be mediated by peripheral eIF3 subunits. However, about 50% of the 80S–IRES complexes lack RACK1, suggesting that this interaction could be transient. Therefore, eIF3 subunits can participate in several steps of IRES-driven initiation of translation.

### Picornavirus IRES Elements

The nucleotide sequences of IRES elements governing translation initiation in the genome of picornaviruses are generally longer than those of dicistrovirus, and include a large number of ignored AUG triplets upstream of the functional start codon, as it also occur on hepacivirus and pestivirus IRES elements. Additionally, picornavirus IRES elements are more heterogeneous in nucleotide sequence, in RNA structure, and in requirement of factors for ribosome recruitment. Because of their large heterogeneity, the IRES elements of picornaviruses are currently classified into five different types. Each type harbors a common RNA structure core maintained by evolutionary conserved covariant substitutions. Nevertheless, the list of new type of IRES elements increases in correlation with the incessant discovery of new species of picornavirus. In addition, recombination events during picornavirus coinfection (Hellen and de Breyne, 2007) can generate IRES elements with unique properties, including novel tissue tropisms and/or host-range spectrum.

**FIGURE 4:**
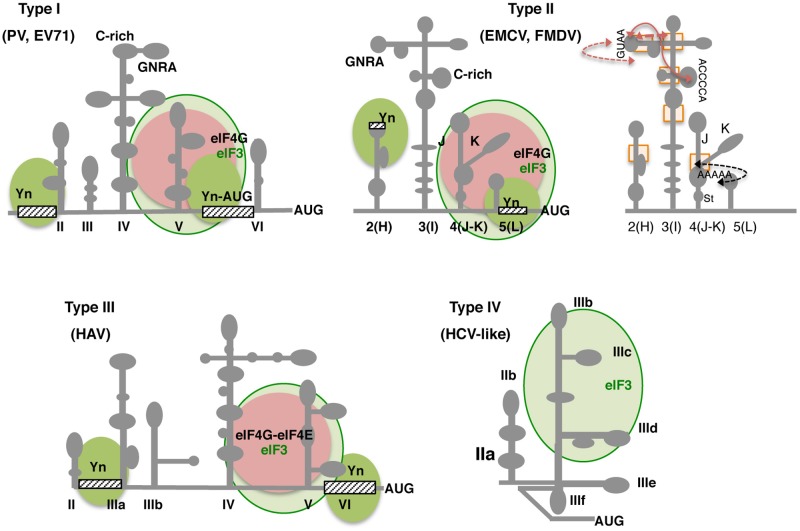
Secondary structure and conserved RNA motifs of picornavirus IRES elements classified as types I, II, III, and IV. Representative members of type I are PV and EV71, members of type II are EMCV and FMDV. Type III is present in HAV, and type IV, also termed HCV-like, is represented by teschovirus IRES. Gray lines depict IRES domains: II–VI in type I; 2 to 5 (or H to L) in type II; II–VI in type III, and IIa to IIIf including a pseudoknot (PK) in type IV. Conserved motifs (GNRA, C-rich, Yn, A-rich) are indicated. Approximate binding sites of eIF3 in all types (light green circle), eIF4G in types I and II, or eIF4G-eIF4E complex in type III (pink circle) are indicated; the recognition of the pyrimidine-rich Yn motif by the polypyrimidine binding protein (PTB) is depicted by solid green circles. Other factors interacting with the IRES are mentioned in the text. For type II IRES elements, brown arrows denote tertiary interactions within the apical region of domain 3; a black arrow depicts the rearrangement of subdomains J-K and stem (St) of domain 4 as a consequence of eIF4G binding (right panel). Rectangles denote regions of local flexibility identified by RNA footprint using dimetallic compounds.

Type I IRES elements occur in the RNA genome of enterovirus [including poliovirus (PV), coxsackievirus B3 (CVB3), enterovirus 71 (EV71), and human rhinovirus (HRV)]. Representative members of type II IRES elements occur in cardiovirus (EMCV) and aphthovirus (FMDV) RNAs. The IRES elements classified as types I and II require the C-terminal region of eIF4G, eIF4A, eIF2, and eIF3 to assemble 48S initiation complexes *in vitro* (Kolupaeva et al., 1998; de Breyne et al., 2009), but are independent of eIF4E. Translation initiation driven by type III, present in the hepatitis A virus (HAV) RNA, was reported to depend on the integrity of eIF4G (Ali et al., 2001). More recent data shows that eIF4E binding to eIF4G generates a high-affinity binding conformation of the eIF4F complex for the IRES (**Figure 4**). Additionally, eIF4E–eIF4G binding stimulates the rate of IRES RNA unwinding by eIF4A (Avanzino et al., 2017). Type IV IRES elements are eIF4G-independent but depend on eIF2 and eIF3. In fact, these group were designated HCV-like (**Figure 4**) because of the similarity with the HCV IRES (Pisarev et al., 2004). A member of a different IRES type is present in the Aichivirus (AiV) genomic RNA. The AiV IRES requires eIF4G, but unlike types I and II, it depends on DHX29, due to sequestration of its initiation codon in a stable hairpin (Yu et al., 2011b).

In the rest of this review, we will concentrate on structural motifs present in types I and II picornavirus IRES elements. Representative members of types I and II IRES elements have been studied in great detail (reviewed in Lozano and Martinez-Salas, 2015). Moreover, due to their generally high efficiency of translation and their complex requirement of factors, these IRES elements are prototypes to study the complexity of mechanisms beyond internal initiation.

Although high-resolution three-dimensional structures of picornavirus IRES elements are still lacking, the secondary structure of picornavirus IRES elements belonging to types I and II has been extensively analyzed (Fernandez et al., 2011a; Yu et al., 2011b; Burrill et al., 2013; Asnani et al., 2015; Lozano et al., 2016a). Type I IRES is about 450 nt long. The RNA structure of the enterovirus IRES element is organized in five domains designated II–VI (**Figure 4**), which adopt stem-loop structures generally including internal bulges (Bailey and Tapprich, 2007). Domain II is a short stem-loop that harbors a conserved internal bulge (AUAGC motif; Levengood et al., 2013). While domains III and VI are rather variable, domains IV and V are conserved. In particular, domain IV (200 nt) adopts a cruciform structure that includes two essential motifs at its apical subdomain, an internal C-rich loop (Gamarnik et al., 2000), and a GNRA (N stands for any nucleotide, and R for purine) tetraloop (Du et al., 2004; Bhattacharyya and Das, 2006). On the other hand, domain V (110 nt) consists of a hairpin with an internal loop. This domain provides the binding site for eIF4G (de Breyne et al., 2009) and for the polypyrimidine tract-binding protein (PTB; Sean et al., 2009). At the 3′ end, a spacer of 18–20 nt separates a non-functional AUG triplet from the conserved pyrimidine tract, about 30–160 nt upstream of the initiation codon. In type I IRES elements, the start codon is selected by scanning (Jackson et al., 2010). However, under certain situations, a direct transfer from the ignored AUG to the functional codon has been reported (Kaminski et al., 2010).

Type II IRES elements are about 450 nt long, and also have a conserved pyrimidine tract upstream of the functional AUG codon at the 3′-border. However, their overall RNA structure differs from type I. The RNA structure of type II IRES is arranged in modular domains designated 2–5 (or H–L, respectively; **Figure 4**; Kaminski et al., 1994; Fernandez et al., 2011b; Lozano et al., 2014). Domain 2 contains a conserved pyrimidine tract that provides a binding site for the PTB protein (Jang and Wimmer, 1990). Domain 3 is a self-folding cruciform structure (Fernandez-Miragall and Martinez-Salas, 2003); the basal region of this domain consists of a long stem interrupted with bulges that includes several non-canonical base pairs and a helical structure essential for IRES activity (Serrano et al., 2009). Domain 4 is organized into two hairpin-loops, which contain the binding site for eIF4G, an essential factor for these IRES elements (Kolupaeva et al., 1998; Lopez de Quinto and Martinez-Salas, 2000; Clark et al., 2003). Domain 5 consists of a phylogenetically conserved hairpin followed by a conserved pyrimidine tract and a variable single-stranded stretch of nucleotides on its 3′ end; this domain provides the binding site for eIF4B and PTB (Ochs et al., 1999; Lopez de Quinto et al., 2001). It is worth noting that a construct that harbors domains 4 and 5 (hence, able to interact with eIF4G, eIF3, eIF4B, and PTB, among other proteins) is not sufficient to promote IRES-dependent protein synthesis (Fernandez-Miragall et al., 2009). These data confirm that although there is a distribution of functions in each domain, the entire element operates as a single entity, since the individual domains are all required for full IRES activity.

Start codon selection on type II IRES elements occurs by direct entry (Jackson et al., 2010). However, a feature of type II IRES elements is the presence of more than one AUG triplet on the initiation zone. In EMCV RNA, the ribosome entry occurs at AUG11, although three AUG triplets (10, 11, and 12) are located in a short window (Kaminski et al., 1994). In FMDV RNA, two in-frame conserved AUGs are separated by a sequence of 84 nt (Belsham, 1992). Deletion of AUG2, but not of AUG1, is detrimental for virus multiplication, consistent with the observation that AUG2 is the preferential initiation site (Lopez de Quinto and Martinez-Salas, 1999). Reconstitution assays with IRES transcripts extended to the second AUG revealed differential requirement of eIF1 to produce a toeprint at AUG2 while eIF1A is required for AUG1. In addition, substitution of AUG1 to AUA does not abrogate protein synthesis, and has no effect on the rate of the 48S complex formation at AUG2, suggesting that the 48S complex formation at AUG2 is independent of AUG1 (Andreev et al., 2007).

Several RNA motifs conserved among type II IRES elements are placed in domain 3, which determines the three-dimensional architecture of the IRES (Ramos and Martinez-Salas, 1999). The apical region of this domain harbors essential motifs for IRES function (Lopez de Quinto and Martinez-Salas, 1997; Robertson et al., 1999). One of the best studied is the GNRA motif, which adopts a tetraloop conformation (Fernandez-Miragall et al., 2006; Dupont and Snoussi, 2009; Mohammed et al., 2014). Computational modeling of this domain generated a three-dimensional RNA structure integrating experimental evidences for tertiary contacts between distant residues of the secondary structure (Jung and Schlick, 2013). These interactions could be transient and do not involve canonical base pairs. On the other hand, it is important to keep in mind that although GNRA tetraloops have been implicated in the establishment of tertiary interactions in RNAs, they can also function as recognition sites for proteins (Thapar et al., 2014). This possibility is under investigation. Noteworthy, both the sequence of motifs exposed on loops, and the sequence of the junctions connecting stems is conserved in field isolates of highly variable RNA viruses, implying that the secondary structure is evolutionary constrained to deliver its function.

Although the function of types I and II IRES elements require eIF4G, the binding site for this canonical eIF have different sequences and structures in the members of these types. The biological relevance of RNA structure for type II IRES elements function was in part supported by the observation of domains 4 and 5 (JKL) rearrangements upon incubation of the EMCV IRES with eIF4G and eIF4A in the presence of ATP (Kolupaeva et al., 2003). These data suggested a change on the mRNA needed to accommodate the initiation codon on the ribosome mRNA cleft. Furthermore, recent data on the structure of the J-K region of the EMCV IRES in solution reveal that stems are precisely organized to position bulges participating in the recognition of proteins. Specifically, a conserved A-pentaloop bulge plays a crucial role as a docking site for base-pair receptors (**Figure 4**). This interaction requires the concerted action of all subdomains, since subtle changes in the orientation abrogate the interaction with eIF4G (Imai et al., 2016). Remarkably, the conformation of the A-pentaloop resembles the GNRA tetraloop, except that the G is substituted by A–A dinucleotide. The similarity of the RNA structure of these conserved motifs opens the question of whether they could derive from repeated RNA modules subjected to genetic changes during evolution, adopting a conformation suitable to acquire novel functions.

RNA flexibility appears to be a key feature of picornavirus IRES elements, as mentioned above for the HCV IRES. In this regard, the implementation of novel di-metallic chemical compounds able to detect flexible regions on the RNA structure in solution have allowed the identification of four-way and three-way junctions within flexible regions of the FMDV and HCV IRES conformation (Lozano et al., 2016b), which are consistent with experimental data obtained from independent approaches (**Figure 4**). Noteworthy, the ability of RNA molecules to acquire distinct conformations in response to specific ligands, as well as environmental signals, determines their function. In addition, conformational transitions could be spatially and/or temporally tuned enabling the assembly of ribonucleoprotein complexes (RNPs) in a hierarchical and sequentially ordered manner. Thus, IRES elements could exploit RNA structure flexibility, and therefore plasticity, as a core functional element.

### Low Structural Complexity IRES Elements

In contrast to the IRES elements described above, the activity of several plant viral IRES elements depends on RNA sequences mostly consisting of single-stranded regions. For instance, the IRES placed upstream of the coat protein ORF of crucifer-infecting tobamovirus (CrTMV) was mapped to A-rich regions (Dorokhov et al., 2006). In this case, mutations in the internal A-tract decrease IRES activity and binding of PABP. Furthermore, enhancement of IRES function in the presence of 3′-poly(A) and the absence of 5′-cap suggest a crosstalk between PABP, the CrTMV IRES and the 3′-poly(A) tail (Marom et al., 2009). Similarly, an unstructured sequence of about 84 nt has been reported to control low levels of translation of the genomic RNA of turnip crinkle virus (TCV) coat protein (May et al., 2017). Surprisingly, in marked contrast to most IRES elements, this A-rich sequence promotes translation irrespectively of the orientation in eIF4G-dependent but in a 4E-independent manner. In another carmovirus, pelargonium flower break virus (PFBV), a region of about 80 nt, which is predicted to be single-stranded, governs the expression of the viral coat protein (Fernandez-Miragall and Hernandez, 2011).

**FIGURE 5:**
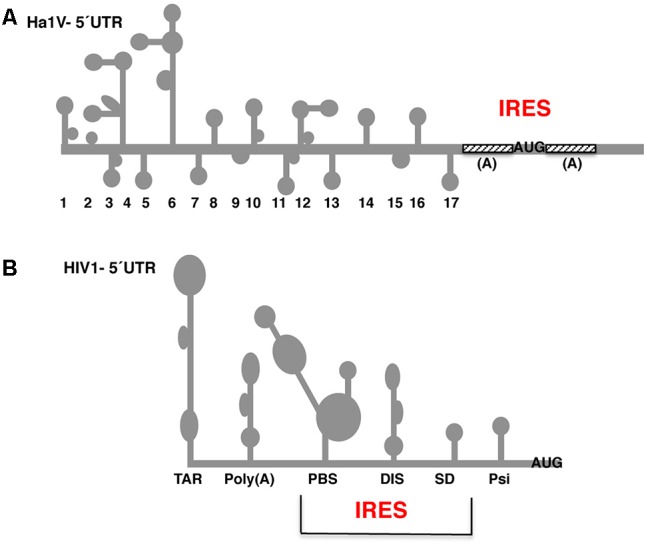
Secondary structure of the 5′ UTR of Halastavi arva virus (HaIV) and HIV-1 RNAs. **(A)** Stem-loops (1–17) of HaIV 5′ UTR are indicated; A-rich unstructured regions flank the functional AUG codon. **(B)** Structural motifs of the HIV-1 5′ UTR are schematically represented. The minimal IRES element overlaps the primer binding site (PBS), dimerization initiation site (DIS), and splice donor (SD) stem-loops. RNA structural motifs within the 5′ UTR flanking the minimal IRES are the trans-activating region (TAR) and the polyadenylation signal (PBS) at the 5′ end, and the packaging signal (Psi) downstream of the IRES element.

A similar type of weakly structured IRES is found in some RNA viruses infecting animal organisms. A representative example of these IRES elements occurs in the A-rich region surrounding the start codon of the Halastavi arva virus (HaIV; **Figure 5A**), a positive-strand RNA virus with a dicistronic genome (Abaeva et al., 2016). In this RNA virus initiation of translation involves unusual direct attachment of the 43S preinitiation complexes immediately downstream of the initiation codon; then, 43S complexes undergo a retrograde scanning dependent on eIF1 and eIF1A. However, the presence of a poly(A) tract on the 5′ UTR of mRNAs is not a distinctive mark of IRES elements, as recently shown for poxvirus mRNAs (Dhungel et al., 2017), which initiate translation by a cap-independent mechanism unrelated to IRES elements.

As opposed to the A-rich unstructured IRES elements, a U-rich single-stranded region determines the activity of a cross-kingdom IRES located at the 5′ end of the dicistrovirus Rhopalosiphum padi virus (RhPV) RNA (Terenin et al., 2005; Groppelli et al., 2007). Ultimately, the diversity in sequence composition and RNA structure organization of IRES elements illustrates the wide variety of possibilities to generate functional RNA regulatory elements.

Collectively, the heterogeneity of IRES structural organization described above points to different strategies developed by viruses to exploit the host translation machinery. At the same, this diversity illustrates the challenges to accurately predict genome-wide the presence of functional IRES elements, or IRES-like elements, in eukaryotic organisms.

### Retrovirus IRES Elements

The 5′ UTR of the genome of retroviruses and lentiviruses adopts a complex secondary structure (Paillart et al., 2004), which in principle should interfere with the cap-dependent mechanism of translation initiation. However, the unspliced capped and polyadenylated viral mRNA is efficiently translated into Gag and Gag-pol proteins. Consistent with the strong secondary structure of the human immunodeficiency virus 1 (HIV-1) 5′ UTR (**Figure 5B**), the RNA helicase DEAD box protein 3 (DDX3) is involved in translation of this atypical mRNA (Soto-Rifo et al., 2013).

Further increasing the diversity of cap-independent translation mechanisms, other studies suggested the presence of IRES-like regions to explain the mechanism of translation initiation in retroviral mRNAs (Herbreteau et al., 2005; Gendron et al., 2008; Vallejos et al., 2010). Similar to other families of RNA viruses, the IRES elements reported in HIV-1 5′ UTR, HIV-1 gag, and HIV-2 gag exhibit different structural features, and also have different requirement of factors. The minimal IRES element of HIV-1 has been mapped overlapping with the primer binding site (PBS), the dimerization initiation site (DIS), and the splice donor (SD) stem-loops (**Figure 5B**). This IRES element is surrounded by other RNA structural motifs, the *trans*-activating region (TAR) and the polyadenylation signal (PBS) at the 5′ end, and the packaging signal (Psi) at the other end (Carvajal et al., 2016). In particular, the HIV-1 5′ UTR IRES tolerates point mutations, but it is strictly dependent on specific host factors and its activity seems to be linked to the cell cycle phase (Vallejos et al., 2011; Soto-Rifo et al., 2012; Carvajal et al., 2016). On the other hand, and in contrast to most viral IRES elements, the HIV-1 gag and HIV-2 gag IRES elements are positioned downstream of the start codon (Locker et al., 2011; Deforges et al., 2017). This characteristic implies a back-scanning mechanism, which is reminiscent of the A-rich mechanism proposed for HaIV IRES (Abaeva et al., 2016).

A common feature of the activity of retrovirus IRES elements is their requirement for eIF5A hypusination (Caceres et al., 2016a). Hypusine is a post-translational modification of eIF5A, which depends on the deoxyhypusine synthase and the deoxyhypusine hydroxylase. Inhibiting activity of the later enzyme, and therefore eIF5A hypusination, reduces HIV-1 IRES activity, and also translation initiation mediated by 5′ UTR of the human T-cell lymphotropic virus type 1 (HTLV-1) and the mouse mammary tumor virus (MMTV) mRNAs.

## Rna Motifs Bridging Rna-Binding Proteins Involved in Ires Function

As mentioned earlier, IRES elements can recruit the ribosomal subunits directly (e.g., by direct contact with the 40S ribosomal subunit), or by using functional bridges, generally eIFs and RBPs. It is well established that eIFs and auxiliary RBPs stimulate 48S complex formation in reconstitution assays using purified factors with picornavirus IRES elements (Andreev et al., 2007; Yu et al., 2011a). Likewise, most IRES transacting-factors (ITAFs) are nuclear proteins that are redistributed to the cell cytoplasm in infected cells (Flather and Semler, 2015; Lee et al., 2017).

**FIGURE 6:**
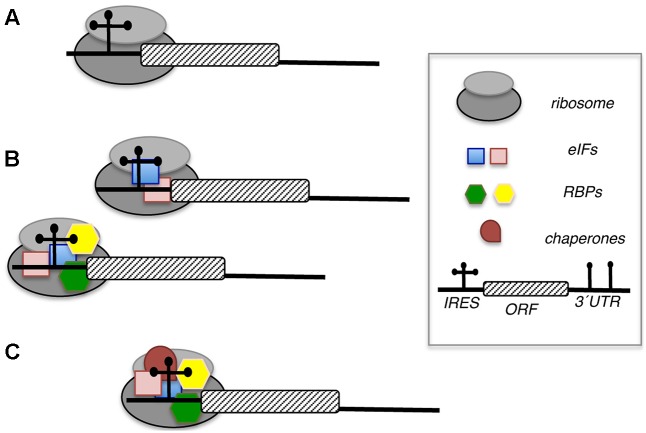
Distinct role of initiation factors (eIFs) and RNA-binding proteins (RBPs) in IRES-dependent translation. **(A)** Direct interaction of the IRES element with the ribosome promotes internal initiation of translation. **(B)** One or more eIFs (blue and pink squares) alone, or eIFs acting in concerted action with several RBPs (green and yellow hexagons) contribute to recruit the ribosome. **(C)** RBPs acting as chaperones (brown circle) stabilize the RNA structure facilitating the interaction with other factors (eIFs or RBPs, square or hexagons, respectively) helping to recruit ribosomal subunits.

The role of RBPs on the activity of viral IRES elements has been studied extensively. Current knowledge on how RBPs modulate IRES function (**Figure 6**) can be summarized in several ways: RNA chaperones stabilizing specific conformations of the IRES structure, stabilizing the interaction of eIFs with the IRES facilitating the recruitment of the ribosomal subunits, mediating a direct recruitment of the ribosome, helping to remove RNA secondary structure near or at the start codon, and/or titrating IRES ligands, either stimulator or repressor molecules. For reasons of space, we will concentrate on RBPs recognizing specific structural motifs of representative members of picornavirus IRES elements and their impact on IRES function.

### Conventional RBPs Modulating IRES Function

A widely conserved RNA motif of viral IRES elements is the polypyrimidine tract, which can vary from 5 to 11 U/C consecutive residues. This motif, which is frequently found in RNAs performing dedicated functions, provides the binding site for PTB (**Figure 4**). Soon after the report of PTB as an EMCV IRES-interacting factor (Jang and Wimmer, 1990), the influence of this protein on internal initiation was analyzed for many other IRES elements (Luz and Beck, 1991; Yu et al., 2011a,b; Caceres et al., 2016b). Interestingly, members of the picornavirus types I and II have two polypyrimidine tracts placed at each end of the IRES region, such that the RNA recognition motifs (RRM) of PTB bind to the IRES constraining the RNA structure in a unique orientation (Kafasla et al., 2009), which finally stimulates protein synthesis.

Another well-studied example of a protein whose role on IRES activity is linked to RNA motifs is poly(C)-binding protein 2 (PCBP2), a factor expressed in most cell types that harbor three ribonucleoprotein K homology (KH) domains. KH domains bind RNA and DNA and are found in proteins controlling transcription and translation, along with other cellular processes. The PCBP2 binding site in the PV IRES is the ACCCC loop, a conserved motif located in domain IV in close proximity to the GNRA tetraloop (Gamarnik et al., 2000). PCBP2 protein is sufficient to complement the activity of canonical eIFs in reconstitution assays with type I IRES elements (Sweeney et al., 2014). Interestingly, the cadicivirus IRES, which contains an essential GNRA tetraloop although it is structurally divergent from type I IRES elements, also require this factor. The KH1 domain of PCBP2 binds near the cadicivirus GNRA tetraloop and is essential for initiation. However, KH3 is critical for PCBP2 binding but not for IRES activity. This observation suggests that PCBP2 could act in concerted action with the GNRA tetraloop to enhance internal initiation. In fact, PCBP2 enhances initiation on mutant IRES elements with a consensus GNRA tetraloop, whereas mutants with divergent sequences do not respond to PCBP2 (Asnani et al., 2016).

A question still unresolved is how the interaction of PCBP2 near the GNRA tetraloop influences the initiation event in type I IRES elements. One possibility could be related to the proximity of these conserved motifs in the three-dimensional structure of type I IRES, reminiscent of the type II IRES elements (**Figure 4**) proposed for the FMDV IRES (Jung and Schlick, 2014). Nevertheless, there are important differences among members of type II. Although the GNRA motif is essential for IRES activity in both EMCV and FMDV IRES (Lopez de Quinto and Martinez-Salas, 1997; Robertson et al., 1999), and despite PCBP2 interacts with both IRES elements, this ITAF is required for EMCV but not for FMDV IRES-dependent translation (Walter et al., 1999). These differences in the biological relevance of RNPs suggest the existence of distinct ways to initiate translation even within members classified within the same type of IRES elements. A second possibility could be that the PCBP2-dependent function of the GNRA tetraloop is linked to the eIF4G/eIF4A-mediated recruitment of the 43S complex (Kolupaeva et al., 2003), because PCBP2 influences eIF4G/eIF4A-mediated conformational changes upstream of the AUG codon. In agreement with the later possibility, the AiV-like IRES is independent of the integrity of the GNRA tetraloop and does not require PCBP2 for initiation, despite possessing a domain IV structure similar to type I (Yu et al., 2011b). In fact, the AiV-like IRES has a type II eIF4G/eIF4A-binding site. However, attachment of 43S complexes to the initiation codon, which is sequestered in a stable hairpin at the 3′-border of the IRES, depends on the helicase DHX29, in contrast to other IRES elements.

Beyond PTB or PCBP2, a relative large number of conventional RBPs involved in a large variety of RNA biology processes occurring both in the nucleus and the cell cytoplasm, have been identified associated with viral IRES elements. Some examples are ErbB3-binding protein 1 (Ebp1, also known as PA2G4 and ITAF_45_), Far upstream element-binding protein 1 (FBP1) and FBP2 (also known as KHSRP, KH-type splicing regulatory protein), Ras GTPase SH3 domain binding protein 1 (G3BP1), heterogeneous nuclear ribonucleoprotein A1 (hnRNPA1), cold-shock domain containing E1 (CSDE1, also known as Unr), or glycil-tRNA synthetase (GARS), to name a few (Monie et al., 2007; Andreev et al., 2012; Hung et al., 2016; Galan et al., 2017; Kung et al., 2017; Tolbert et al., 2017). Furthermore, these factors can modulate IRES activity in a positive or negative manner (for a recent review, see Lee et al., 2017). Most of these proteins interact with multiple targets that, in turn, are members of RNP networks that may respond in a coordinated manner to changes in the cell environment. The relative low specificity of RNA-binding factors interacting with IRES elements represents an obstacle to discriminate if the proteins recognizing distinct IRES elements reflect common evolutionary traits. In turn, this hinders the possibility to discriminate whether this is a result from promiscuous binding to RNA, or if it is the consequence of forming part of networks involved in RNA-dependent processes. Not surprisingly, RNP networks regulating distinct RNA-dependent processes share many components (Gehring et al., 2017).

### Novel Actors in the Translation Control Scenario

A different example of a host factor recognizing a structural motif involved in internal initiation is Gemin5. Similar to other IRES-binding factors, this protein is involved in several steps of the RNA metabolism, but in contrast to PTB or PCBP2, negatively modulates IRES activity (Pacheco et al., 2009). This multifunctional protein has a role on translation control that relies on its capacity to recognize different RNAs (Workman et al., 2015). Gemin5 is a predominant cytoplasmic protein that has two distinct functional domains. At the N-terminus, a 14 WD40 repeat motif domain is responsible for the delivery of the spinal motor neuron complex to snRNAs (Xu et al., 2016), in addition to provide a platform for protein–protein interactions. In fact, proteins identified as Gemin5 N-terminal moiety associated factors include RBPs, ITAFs and ribosomal proteins, among many others (Francisco-Velilla et al., 2016). In contrast, the C-terminal domain mediates the interaction with the FMDV IRES element (Pineiro et al., 2013). The ribosome-binding capacity of the N-terminal moiety enables Gemin5 to control global protein synthesis (Francisco-Velilla et al., 2016), while the non-canonical RNA-binding domain located at the C-terminal end is responsible for the negative effect on IRES-dependent translation (Fernandez-Chamorro et al., 2014). Notwithstanding, Gemin5 is proteolyzed in infected cells by the action of the FMDV L protease (Pineiro et al., 2012). Consistent with the negative effect of this protein on translation, the cleavage product corresponding to the C-terminal domain is not stable in infected cells, while the precursor p85, that exhibits a translation stimulatory effect, is readily immunodetected in cell lysates through infection (Pineiro et al., 2012).

Besides cleavage by viral proteases (reviewed in Walsh and Mohr, 2011; Flather and Semler, 2015; Martinez-Salas et al., 2015 and references therein), several post-translational modifications of RBPs affect their activity on IRES-dependent translation. For instance, an IRES-binding protein that is specifically modified during infection is the KH domain of nuclear protein 68-kDa Src-associated in mitosis (Sam68). This protein is methylated and redistributed from the nucleus to the cell cytoplasm in FMDV infected cells (Lawrence et al., 2012), leading to the stimulation of IRES-dependent activity (Rai et al., 2015). In addition, Sam68 recognizes stem-loops IV and V of the EV71 IRES region, and regulates IRES-dependent translation (Zhang et al., 2015). Likewise, Sam68 is redistributed from the nucleus to the cytoplasm during EV71 infection, and it has been proposed that its interaction with PCBP2 and PABP may be involved in the enhancement of EV71 IRES-mediated translation. This view is consistent with contingency ideas of a macromolecular RNP network controlling IRES function. An important point to keep in mind is that not all IRES elements are equally active, and also that IRES activity depends not only on the cell type but also on the cellular environment, reflecting the availability of RBPs and other RNA cofactors in the cell cytoplasm.

### Stimulation of IRES Function by Distant RNA Regions

RNA virus genomes fold into complex structures that include long-range RNA–RNA interactions relevant to control critical steps of the viral cycle (for recent reviews on this topic, see Nicholson and White, 2014; Jan et al., 2016). In particular, initiation of translation driven by the IRES elements of FMDV and HCV is stimulated by sequences located on the 3′ end of the viral RNA (Lopez de Quinto et al., 2002; Song et al., 2006; Romero-Lopez and Berzal-Herranz, 2009; Garcia-Nunez et al., 2014). Initially, long-distant RNA–RNA interactions on the FMDV genome were supported by gel mobility-shift data obtained *in vitro* using specific transcripts (Serrano et al., 2006). More recently, the implementation of *in vivo* Selective 2′-Hydroxyl Acylation Analyzed by Primer Extension (SHAPE) methodologies to analyze the RNA local flexibility of the FMDV IRES and the 3′ UTR in living cells confirmed the existence of long-range interactions (Diaz-Toledano et al., 2017). In comparison to a transcript that lacked the 3′ UTR, statistically significant decrease of reactivity was observed at IRES residues placed immediately upstream from the functional start codon. Conversely, presence of the IRES element *in cis* altered the 3′ UTR local flexibility leading to overall enhanced reactivity. Unlike the reactivity changes observed in the IRES element, the SHAPE differences of the 3′ UTR were not statistically significant, suggesting a dynamic RNA structure. Covariation analysis predicted IRES-3′ UTR conserved helices in agreement with the protections observed by SHAPE probing. In support of this notion, engineering alternative base pair interactions compensated experimental disruption of the predicted base pairs. These results provided direct evidences for dynamic long-range interactions between these distant elements of the viral genome.

Long-range interactions in viral RNAs controlling fundamental processes of the viral replication cycle, such as translation and replication, have been extensively documented. Readers are directed to recent reviews discussing examples on cap-independent translation enhancers and other RNA-looping examples (Simon and Miller, 2013; Nicholson and White, 2014; Jan et al., 2016).

## Conserved Rna Motifs: a Therapeutic Target and a Hint to Predict Ires-Like Motifs

RNA viruses have small, compact genomes. Thus, expression of their genome strongly depends on the host cell. This feature ensures that viral genomes evolved to increase their replication potential in the infected host, generally using unique mechanisms usurping the cellular machinery in their own benefit. In turn, this characteristic provides useful therapeutic targets. Because IRES elements are essential for viral infection and harbor conserved RNA motifs, they constitute specific targets for antiviral compounds, as exemplified by a large variety of modified antisense oligonucleotides, siRNAs, morpholino oligomers, aptamers, and small molecules based on benzimidazole (Aldaz-Carroll et al., 2002; Vagnozzi et al., 2007; Stone et al., 2008; Laxton et al., 2011; Fajardo et al., 2012; Boerneke et al., 2014; Sanchez-Luque et al., 2014; Lozano et al., 2015).

The evolutionary plasticity of RNA viruses makes them an ideal system for identifying mechanisms used not only by viruses, but also by the host cell. The observation that IRES-like sequences were described in cellular RNAs (Thakor and Holcik, 2012; Karginov et al., 2016) provides support for the idea that these elements may have appeared several times during evolution. Early studies on the mechanism of action of IRES elements suggested similarities with prokaryote-like mechanisms (Pestova et al., 1998). In agreement with the possibility that internal initiation could be at least in part mediated by direct interactions of the IRES with the ribosomal RNA, the study of the cellular IRES located on the insulin-like growth factor 1 receptor (IGF1R) mRNA proposed a direct interaction with the 18S rRNA modulated by a U-rich loop (Meng et al., 2010), complementary to a solvent accessible loop close to the E-site (nt 950–974) of the ribosome (Ben-Shem et al., 2011). Moreover, in spite of the fact that earlier work reported the need of eIFs for the *in vitro* assembly of 48S complexes with picornavirus type II IRES elements (Pestova et al., 1996), direct interactions between the 40S ribosomal subunit and the EMCV IRES have been described (Chamond et al., 2014).

Given the diversity of IRES-driven mechanisms to govern protein synthesis among viral RNAs, it is also conceivable that some type of IRES elements could arise by convergent evolution. Possible events resulting in the generation of novel IRES elements could be the assembly of RNA modules derived from different molecules by RNA recombination, integrative events, or RNA ligation. These events, although infrequent, could render structural elements with functions unrelated to the RNA molecules harboring the original modules. Indeed, RNA molecules have unique structural attributes, which include the ability to self-assemble through the arrangement of building blocks (Grabow and Jaeger, 2014). An example of a structural motif conserved in various RNA viruses is the tRNA-like motif (Dreher, 2009). Interestingly, tRNA-like motifs promote cap-independent translation in the genome of plant RNA viruses (Le et al., 2017). Moreover, tRNA-like signals have been suggested to be present in viral IRES elements (Piron et al., 2005; Serrano et al., 2007), and proteins involved in tRNA metabolism have been related to IRES-dependent translation (Andreev et al., 2012).

The relationship between RNA structure and biological function is generally inferred from conservation of structural motifs. Because RNA structure plays a fundamental role in most viral IRES elements (Lozano and Martinez-Salas, 2015), it is plausible that conserved motifs present in viral IRES elements would be advantageous to search for regions putatively folding as IRES-like subdomains in genome sequences. In turn, this could provide hints about their evolutionary history, in addition to expand our understanding of gene regulatory elements. Accurate prediction of IRES elements is currently a challenging task (Kolekar et al., 2016). Notwithstanding, the unusual combination of conserved motifs within the apical region of domain 3 of type II IRES elements was the basis for a computational search via RNA inverse folding (Dotu et al., 2013), putatively adopting an IRES-like subdomain fold. Subsequent application of filters and biological insights to prioritize the hits returned, this approach predicted a reduced number of cellular mRNA sequences adopting an IRES-like subdomain. Future studies using RNA inverse folding approaches (Garcia-Martin et al., 2016; Churkin et al., 2017) could facilitate the search for other structural conserved motifs present in prototype viral IRES elements. Along this idea, it is rather unlikely that only RNA viruses take advantage of the IRES-dependent translation initiation mechanism. Cap-independent translation occurs during a large variety of physiological cellular processes, such us apoptosis, osmotic stress, nutrient deprivation, or cell differentiation, not only when the cap-dependent translation machinery is compromised by viral infection (Jan et al., 2016). Moreover, the recent discovery of novel polycistronic monopartite viral RNAs comprising five ORFs (Olendraite et al., 2017) strongly suggest that internal initiation could be at the basis of gene expression in many still unknown organisms. Therefore, it is plausible that IRES elements could have appeared several times during evolution, thus explaining the large diversity of sequences resulting in internal initiation of translation.

## Concluding Remarks

In this review, we have discussed examples of conserved RNA motifs impacting on a different extent on the activity of viral IRES elements. The influence of conserved motifs could be due to their impact on the IRES structure organization, but also on the influence on RBPs recognition and ribosome recruitment. Furthermore, although IRES elements from genetically distant viral RNAs lack overall conserved features, most of them have RNA structure flexibility. This characteristic increases in correlation with the requirements of factors to assemble competent translation initiation complexes. In this regard, it is important to note that the functional features of RNA molecules are established in their three-dimensional structure, but also in their ability to acquire distinct conformations in response to specific signals. In addition, conformational transitions could be spatially and temporally tuned to achieve distinct functions enabling assembly of RNPs in a hierarchical and sequential ordered manner. Thus, IRES elements could exploit RNA structure flexibility, and thus plasticity, as a core functional element. Future studies aimed to understand the structural organization and function of diverse viral IRES elements will help to improve the accuracy to predict IRES-like motifs in other genomes, regardless of the lack of a universal structural motif unique to all viral IRES elements.

## Author Contributions

All authors listed have made a substantial, direct and intellectual contribution to the work, and approved it for publication.

## Conflict of Interest Statement

The authors declare that the research was conducted in the absence of any commercial or financial relationships that could be construed as a potential conflict of interest.
